# Enhanced Ferromagnetic Interaction in Modulation-Doped GaMnN Nanorods

**DOI:** 10.1186/s11671-017-2061-5

**Published:** 2017-04-20

**Authors:** Yuan-Ting Lin, Paritosh Vilas Wadekar, Hsiang-Shun Kao, Yu-Jung Zheng, Quark Yung-Sung Chen, Hui-Chun Huang, Cheng-Maw Cheng, New-Jin Ho, Li-Wei Tu

**Affiliations:** 1 Department of Physics and Center for Nanoscience and Nanotechnology, National Sun Yat-Sen University, Kaohsiung, 80424 Taiwan, Republic of China; 2 Department of Materials and Optoelectronic Science and Center for Nanoscience and Nanotechnology, National Sun Yat-Sen University, Kaohsiung, 80424 Taiwan, Republic of China; 3 National Synchrotron Radiation Research Center, Hsinchu, 30076 Taiwan, Republic of China; 4 Department of Medical Laboratory Science and Biotechnology, Kaohsiung Medical University, Kaohsiung, 80708 Taiwan, Republic of China

**Keywords:** A1. Characterization, A1. Doping, A3. Molecular beam epitaxy, B1. Nanomaterials, B1. Nitrides, B2. Magnetic materials

## Abstract

In this report, ferromagnetic interactions in modulation-doped GaMnN nanorods grown on Si (111) substrate by plasma-assisted molecular beam epitaxy are investigated with the prospect of achieving a room temperature ferromagnetic semiconductor. Our results indicate the thickness of GaN layer in each GaN/MnN pair, as well as Mn-doping levels, are essential for suppressing secondary phases as well as enhancing the magnetic moment. For these optimized samples, structural analysis by high-resolution X-ray diffractometry and Raman spectroscopy verifies single-crystalline modulation-doped GaMnN nanorods with Ga sites substituted by Mn atoms. Energy dispersive X-ray spectrometry shows that the average Mn concentration can be raised from 0.4 to 1.8% by increasing Mn fluxes without formation of secondary phases resulted in a notable enhancement of the saturation magnetization as well as coercive force in these nanorods.

## Background

Combining spin and charge functionalities is expected to bring a renaissance in compound semiconductors. Diluted magnetic semiconductors (DMSs) are one group of compounds where such possibilities are feasible [1]. Among potential candidates, there are group III nitrides that are widely used in commercial optoelectronic devices, such as light-emitting diodes (LEDs) and laser diodes (LDs). By enabling spin functionality, one could envision the creation of new devices or enhanced functionalities; hence, obtaining above-room-temperature ferromagnetism in nitride semiconductors is an important topic of scientific research [2]. Besides this, III nitride can be grown into nanostructures as nanocolumn LEDs and nanowire photodetector and quantum dot lasers as potential next-generation devices [3–5]. Thus, studying the possibility of incorporating spin functionality in such nanostructures has been a major goal in the scientific community [6–12]. Typically, growth of one-dimensional nanostructures such as nanowires is readily achieved by chemical vapor deposition (CVD) [11, 13, 14], but a proper ordering of nanostructures, necessary for device fabrication, is lacking unless some extra fabrication steps are used [15]. An alternative to CVD growth is the self-assembly methodology of nanorods using plasma-assisted molecular beam epitaxy (PAMBE, Veeco) wherein no additional fabrication steps are required [16–18]. Our previous results show that it is indeed possible to incorporate Mn into GaN for creating nanostructures that exhibit room temperature ferromagnetism by incorporating a modulation-doping approach wherein a thin Mn layer is sandwiched between a thicker GaN spacer [19].

One major bottleneck for superior devices is the need for stronger magnetic signals such as saturation magnetization, remnant magnetization, and coercive force. Since the ferromagnetic properties of semiconductors depend on the amount of magnetic dopant, it is challenging to prepare thin films without the formation of any secondary phases such as ferromagnetic nanoclusters that might result in a spurious signal in magnetic measurements. Furthermore, the itinerary carriers in the host semiconductor have to couple with the localized magnetic moment and enhance the carrier-mediated ferromagnetic properties based on double exchange mechanism. The ferromagnetism of GaN-based DMSs has been reported to relate closely with the type and concentration of activated carriers [20, 21]. Considering all above, modulation-doping technique is adopted to obtain a high dopant concentration and carrier concentration in one or few monolayers [22]. The magnetic properties of GaN-based DMSs have been reported to be enhanced in GaMnN/GaN multilayers and GaGdN/GaN superlattice structures as compared to a single layer with normal doping method [20, 23]. In this report, we have fabricated self-aligned phase pure vertical nanorods which exhibit  superior ferromagnetic properties beyond room temperature. Our investigations reveal that the amount of Mn dopant and GaN spacer thickness are crucial for the enhancement of the ferromagnetic properties as well as avoiding secondary phase formation.

## Methods

GaN nanorods were grown by plasma-assisted molecular beam epitaxy (Veeco 930) on Si (111) substrate without a buffer layer. The Si wafer was chemically cleaned before loading into the chamber and thermally cleaned after loading into the chamber as usual [19]. The growth sequence was to grow undoped GaN nanorod section first as a template, and then, modulation-doped GaMnN was grown on top of the undoped nanorod section to complete the nanorod growth. It retained the shape and vertical directions of the bottom undoped nanorod template. The modulation-doping technique utilized the metal modulation method, that is, the Mn-flux and Ga-flux modulations were achieved by controlling the open and close of the shutters of Mn and Ga while keeping the nitrogen flow uninterrupted. The metallic sources (Mn, Ga) were provided through Knudsen effusion cells and controlled by fast-action pneumatically driven shutters in front of the cells. The active nitrogen species were supplied by the radio-frequency UNI-Bulb plasma source at a fixed power of 450 W.

To study the growth of Mn modulation-doped GaN nanorods, there are two series of samples grown in this report as shown in Table 1. The substrate temperature was kept at *T*_s_ = 700 °C for both series of samples. In the first series of modulation-doped GaMnN nanorods, series I, GaN growth was interrupted 300 times through a 2-s duration of Mn flux. The thickness of GaN spacer in each pair was varied and controlled by its growth time with 5, 10, 20, and 40 s with an estimated growth rate of 0.09 nm/s. In the second series, series II, the Mn flux was raised further from 1.7 × 10^−8^ to 2.4 × 10^−8^ Torr to increase the Mn concentration of GaMnN nanorods with a total GaN/MnN growth period of 180.

**Table 1:** Sample growth parameters and Mn concentration

	Sample	GaN/MnN pairs	Growth time per pair of GaN/MnN (s)	Mn flux (10^−8^ Torr)	EDS percentage of Mn (atomic%)
Series I	5s-MnNR	300	5/2	1.7	0.8
	10s-MnNR	300	10/2	1.7	0.7
	20s-MnNR	300	20/2	1.7	0.6
	40s-MnNR	300	40/2	1.7	0.4
Series II	S-MnNR	180	40/2	1.7	0.7
	M-MnNR	180	40/2	1.9	1.8
	L-MnNR	180	40/2	2.4	3.3

The morphology and structure of nanorods were inspected by field emission scanning electron microscopy (FESEM, JEOL JSM-7000F) and high-resolution transmission electron microscopy (HRTEM, FEI Tecnai G2 F20). The concentration of Mn in GaMnN nanorods was examined by the energy dispersive X-ray spectrometry (EDS, Oxford INCA Penta FETX3) installed in the FESEM system. The phase purity of Mn modulation-doped GaN nanorods and the local structure of Mn atoms in GaN host lattice were studied by high-resolution X-ray diffractometry (HRXRD, Bede D1) and Raman spectroscopy (Jobin Yvon T64000), respectively. Furthermore, magnetic properties of GaMnN nanorods at above room temperature were carried out by using a superconducting quantum interference device (SQUID) magnetometer (Quantum Design MPMS-XL7). X-ray absorption near edge spectra (XANES) measurements of N K-edge, Ga L-edge, and Mn L-edge was performed at BL08B1 and BL20A1 beamlines of the National Synchrotron Radiation Research Center (NSRRC) in Hsinchu, Taiwan. All N K-edge, Ga L-edge, and Mn L-edge spectra were recorded at the incident beam with E-field polarization parallel to the surface of the sample at room temperature by total electron yield (TEY) mode. XANES of Mn L-edge from a MnO single crystal was taken simultaneously for the energy calibration and the comparison of different electronic valence states.

## Results and Discussion

**Fig. 1:**
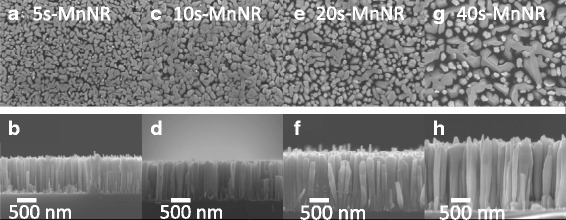
The top (**a**, **c**, **e**, **g**) and cross-sectional (**b**, **d**, **f**, **h**) view SEM images of modulation-doped GaMnN nanorods with GaN growth time in each pair as 5 s (5s-MnNR), 10 s (10s-MnNR), 20 s (20s-MnNR), and 40 s (40s-MnNR), respectively

**Fig. 2:**
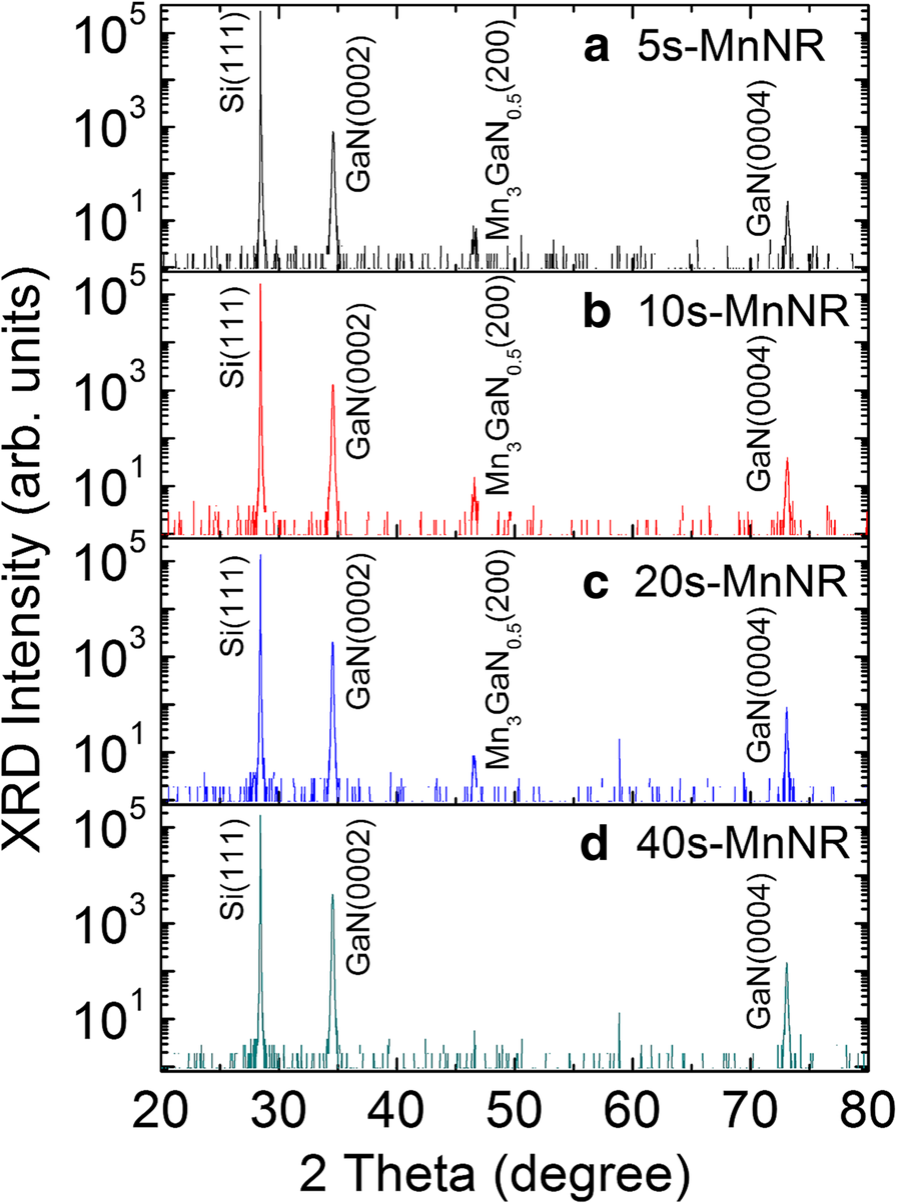
**a**–**d** HRXRD spectra of modulation-doped GaMnN nanorods with varied growth time of GaN. GaMnN nanorods with Mn-related secondary phases are observed except the sample grown with 40-s growth time of GaN spacer (40s-MnNR in **d**)

The top and cross-sectional view of FESEM images of the modulation-doped GaMnN nanorods with 5-, 10-, 20-, and 40-s growth time of GaN spacer in GaN/MnN pair structure (labeled as samples 5s-MnNR, 10s-MnNR, 20s-MnNR, and 40s-MnNR, respectively) are shown in Fig. 1a–h, respectively. The height of these samples is 750, 850, 1200, and 1700 nm, for 5s-MnNR, 10s-MnNR, 20s-MnNR, and 40s-MnNR, respectively. The GaN spacer thickness in each pair can be estimated by the average growth rate of the nanorods, which is 0.09 nm/s. Moreover, inclinations on the top of Mn-doped GaN nanorods are observed in cross-sectional SEM image. Figure 2 shows the HRXRD spectra of modulation-doped GaMnN nanorods for the series I. The peaks at 28.44°, 34.56°, 58.88°, and 72.90° in *θ*/2*θ* scan are referred to cubic Si (111), wurtzite GaN (0002), Si (222), and GaN (0004), respectively, which also indicate that nanorods are grown along the *c*-axis of a wurtzite structure. This wide range X-ray diffraction scan shows that there is no obvious secondary phase in sample 40s-MnNR with about 3.6 nm GaN spacer in each pair. However, an additional peak at 46.58°, which is referred to Mn_3_GaN_0.5_ (200) [24], is observed in GaMnN nanorods while the growth time of GaN is equal to or less than 20 s. The formation of this Mn-rich secondary phase is explained by the higher chance of more Mn ions aggregated together with the thinner GaN spacer. Mn atoms have also been proven to escape from a modulation-doped layer to the surface over a GaN layer of fewer than 11 monolayers in an MBE grown thin film at 670 °C [25].

**Fig. 3:**
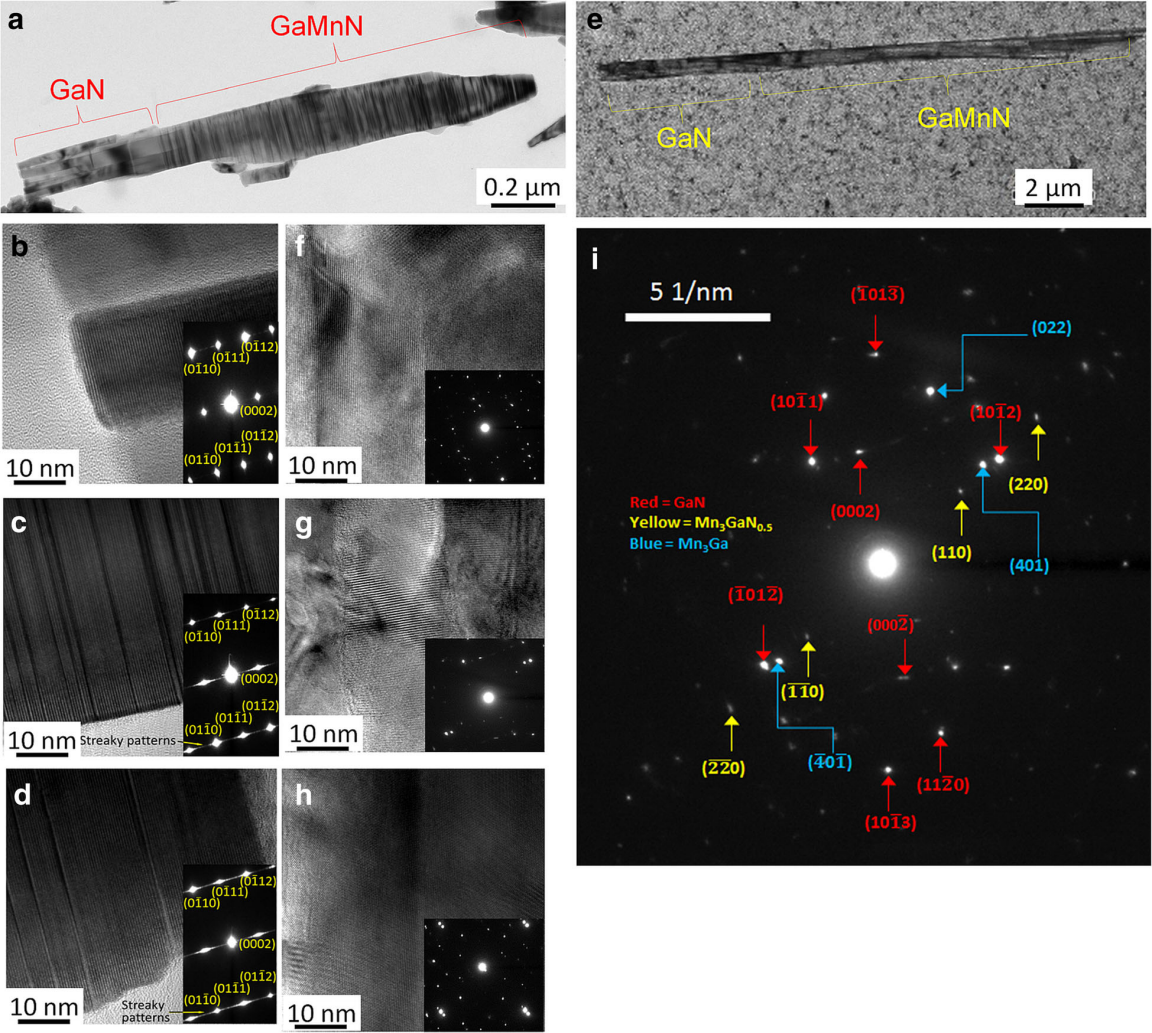
**a** Low-magnification transmission electron microscopy images of the nanorod sample 40s-MnNR. **b**, **c**, **d** The HR images for the bottom, middle, and top regions of the nanorod sample. The diffraction spots are in the *inset* for the respective regions. **e** Low-magnification transmission electron microscopy images of the nanorod sample L-MnNR. **f**, **g**, **h** The HR images for the bottom, middle, and top regions of the nanorod sample. The diffraction spots are in the *inset* for the respective regions. **i** Indexed patterns for the middle region of the L-MnNR

The microstructure of sample 40s-MnNR was studied using high-resolution TEM by measuring atomic images. Figure 3a shows a low-magnification image. The bottom region, marked as GaN, is quite clear while the top region marked as GaMnN region shows an alternation of dark and bright layers lying parallel along the nanorod diameter. To further understand the crystal structure, selected area electron diffraction (SAED) measurements were done at different positions of the nanorod. Figure 3b–d shows the SAED for the bottom, middle, and top regions of the nanorod along with the appropriate indexing. The close resemblance of the diffraction patterns indicates that integrity of the crystal structure is maintained even as Mn is incorporated. A closer inspection of the diffraction patterns in Fig. 3c, d shows some fine features such as streaking along the [0001] axis. The presence of such streaking is due to the formation of stacking faults [19]. The diffraction analysis of the sample 40s-MnNR does not show any secondary phases, collaborating with the XRD data. The Mn contents in modulation-doped GaMnN nanorods are evaluated by the EDS equipped in the SEM system as shown in Table 1. As a result, the average Mn contents of samples 5s-MnNR, 10s-MnNR, 20s-MnNR, and 40s-MnNR are read as 0.8, 0.7, 0.6, and 0.4%, respectively. The Mn concentration is reduced with the increasing growth time of GaN. This is attributed to the dilution of the average Mn concentration by the increased thickness of GaN in a GaN/MnN periodical structure.

**Fig. 4:**
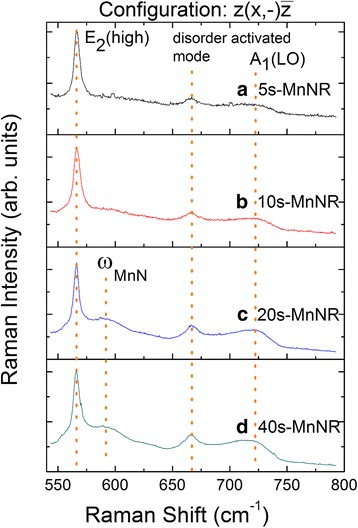
**a**–**d** Raman Stokes spectra of modulation-doped GaMnN nanorods with varied growth time of GaN. The local vibration mode of Mn occupying the Ga site (ω_MnN_) is observed at 588 cm^−1^ in modulation-doped GaMnN nanorods

The role of Mn atoms in GaMnN nanorods is investigated by Raman spectroscopy at room temperature with Stokes shifts as shown in Fig. 4. All samples show the clear E_2_(high) mode of a wurtzite GaN at 567 cm^−1^. The presence of frequency ω_MnN_ at 588 cm^−1^ is assigned to the local vibration mode arising from Mn atoms occupying the Ga sites, which is comparable to the value of 581 cm^−1^ calculated from a simple mass defect model [19, 26]. Nevertheless, the samples 5s-MnNR and 10s-MnNR, which contain higher average Mn concentration, do not reveal clear ω_MnN_ signal. This result indicates that less Mn atoms substitute Ga atoms in GaMnN, which is consistent with the Mn-related secondary phase revealed by XRD data in GaMnN nanorods with GaN growth time equal to or less than 20 s. Furthermore, the fairly constant signal at 670 cm^−1^ is ascribed to disorder activated Raman scattering of vacancy-related defects [27]. The A_1_(LO) signal at 725 cm^−1^ arises with the ω_MnN_ signal. It indicates a low free carrier concentration in Mn-substituted samples [28]. This can be explained by the formation of a deep level within the GaN bandgap. Many reports on optical measurements reveal that Mn-doped GaN would create an acceptor level of 1.4–1.8 eV above the valence band [29, 30].

**Fig. 5:**
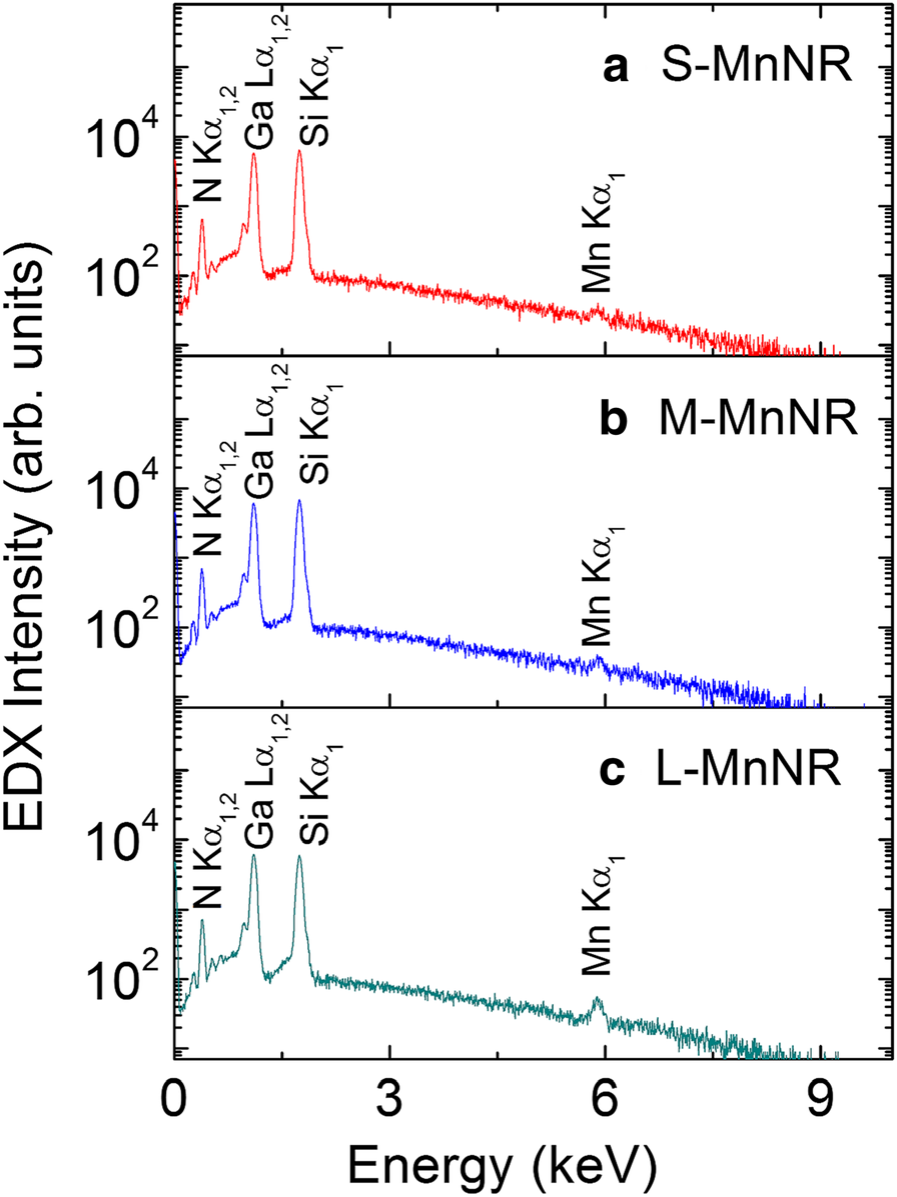
EDS spectra of modulation-doped GaMnN nanorods with different Mn fluxes. The average Mn content of **a** S-MnNR, **b** M-MnNR, and **c** L-MnNR are 0.7, 1.8, and 2.4%, respectively

**Fig. 6:**
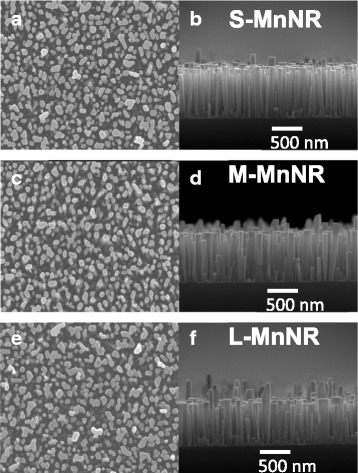
SEM top view (**a**, **c**, **e**) and cross-sectional view images (**b**, **d**, **f**) for modulation-doped GaMnN nanorods with different Mn fluxes

To further increase the Mn concentration and hopefully increase the ferromagnetic properties, a second series, series II, of samples of modulation-doped GaMnN nanorods with varied Mn fluxes was grown. According to the results of series I, the secondary phases in modulation-doped GaMnN nanorods can be suppressed by a GaN barrier with 3.6 nm in thickness in each pair. Hence, the growth time of GaN/MnN in series II was fixed to 40/2 s, that is, the Ga shutter opens 40 s and the Mn 2 s for each period. Modulation-doped GaMnN nanorods of samples S-MnNR, M-MnNR, and L-MnNR were grown with Mn fluxes of 1.7 × 10^−8^, 1.9 × 10^−8^, and 2.4 × 10^−8^ Torr, respectively. The V/III ratios were raised from 500 in series I to 1000 to further enhance the incorporation of Mn comparing samples 40s-MnNR and S-MnNR as displayed in Table 1. Figure 5 shows the EDS spectra of modulation-doped GaMnN nanorods with different Mn fluxes measured from the top of the samples. The signals of N K_α_, Ga L_α_, Si K_α_, and Mn K_α_ are observed at 0.39, 1.10, 1.74, and 5.90 keV, respectively. It is noticed that the peak intensity of Mn K_α_ is enhanced with the increasing Mn flux. Furthermore, the average Mn contents of S-MnNR, M-MnNR, and L-MnNR from EDS measurements are summarized in Table 1 as 0.7, 1.8, and 3.3%, respectively. Figure 6a–f shows the top and cross-sectional view of the samples S-MnNR, M-MnNR, and L-MnNR, respectively. A higher V/III ratio increases the separation of nanorod structure. With increasing Mn fluxes, the uniformity in its morphology is decreased. The GaMnN nanorods with varied Mn fluxes have similar density and about 700 nm height, as shown in cross-sectional SEM images. A little lower in height than what is expected from series I indicates possibly a little lower nitrogen plasma efficiency. All three samples show the ω_MnN_ vibration mode in Raman spectroscopy indicating the success replacement of Ga by Mn.

**Fig. 7:**
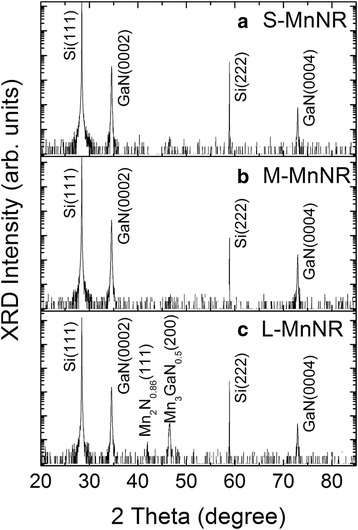
**a**–**c** HRXRD spectra of modulation-doped GaMnN nanorods with different Mn fluxes show single-crystalline GaMnN nanorods without observable secondary phase except the GaMnN nanorods having the highest Mn content, L-MnNR in **c**

The XRD *θ*/2*θ* spectra of modulation-doped GaMnN nanorods with different Mn fluxes are shown in Fig. 7. Like the samples in series I, single-crystalline GaMnN nanorods grown on Si (111) substrate along *c*-axis show main peaks at 28.44°, 34.56°, 58.88°, and 72.90° referred to cubic Si (111), wurtzite GaN (0002), Si (222), and GaN (0004) diffractions, respectively. However, the secondary phases of Mn_2_N_0.86_ (111) and Mn_3_GaN_0.5_ (200) are observed at 42.00° and 46.58° in heavily Mn-doped GaN nanorod, sample L-MnNR [31, 32], which indicates that the average 3.3% Mn concentration has exceeded the solubility of Mn in these GaN nanorods to form Ga_1 − *x*_Mn_*x*_N with Mn substituting the Ga under the growth conditions. In comparison, MBE grown GaMnN thin films with Mn concentration higher than 6% and up to 8.2% have been reported [33, 34]. Lower solubility of Mn in GaN nanorods can be attributed to the higher growth temperature [35, 36]. Mn-related precipitates are more easily formed in MBE growth with increased temperature [37]. The other possibility is the higher than average value near the MnN layers in these samples which are then averaged out by the GaN layers. We have used HRTEM to identify if such secondary phases can be observed. Figure 3e shows the low-magnification imaging for the multiphase L-MnNR sample while Fig. 3f–h shows the atomic imaging for the different regions. Comparing the multiphase sample to the single-phase sample 40s-MnNR in Fig. 3a–d, the layering in single-phase sample is absent in the multiphase one. The SAED patterns from various regions of the multiphase sample are also shown in the insets which show many diffraction spots. It is difficult to find one periodic ordering that will successfully index all the spots. We selected the diffraction pattern from the middle region which is less complex as compared to the other two and tried to identify the various phases present as in Fig. 3i. By measuring the distance from the center spot to the other spots, we calculated the reciprocal lattice vectors and compared them to known values from literature for GaN, GaMn, and Ga-Mn-N compounds. Some of the bright spots can be indexed to wurtzite GaN lying along the *m*-zone $\left(10\overline{1}\mathrm{l}\right)$ and *c*-zone (000l) (labeled in red). The other strong set of spots was indexed to orthorhombic Mn_3_Ga (labeled in blue), while the weak spots were indexed to Mn_3_GaN_0.5_ (labeled in yellow). So while it is difficult to image a nanocluster in the imaging mode, SAED measurements can be useful for the analysis.

**Fig. 8:**
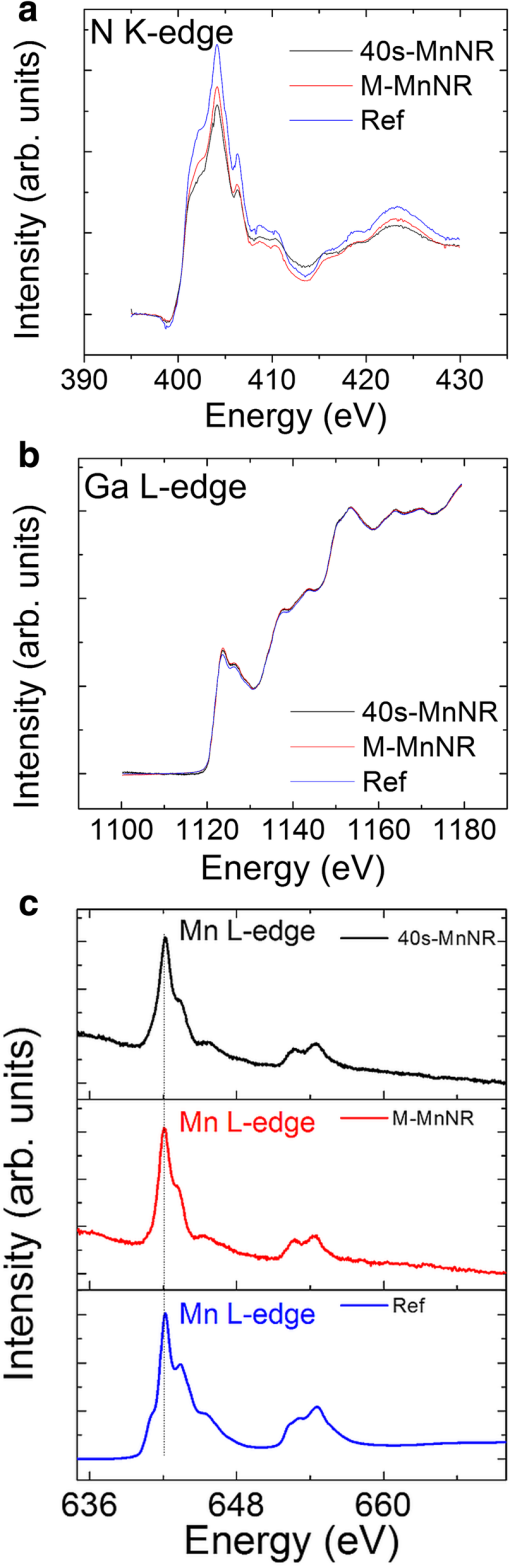
XANES spectra for N K-edge, Ga L-edge, and Mn L-edge are plotted in **a**, **b**, and **c**, respectively. Reference sample in **a** and **b** is undoped GaN nanorod sample and in **c** MnO single crystal

We have studied the electronic properties of the single-phase samples by XANES. The various spectra are plotted in Fig. 8a–c for N K-edge, Ga L-edge, and Mn L-edge, respectively. As seen, no obvious differences in the Ga or the N signal are seen for the Mn-doped samples as compared to the reference undoped GaN nanorod sample implying that that local electronic structure is the same. The valence state of Mn is +2 as judged by the same peak shapes in the Mn-doped samples as compared to the reference sample which is MnO single crystal and with other reported results for Mn-doped GaN [38–40].

**Fig. 9:**
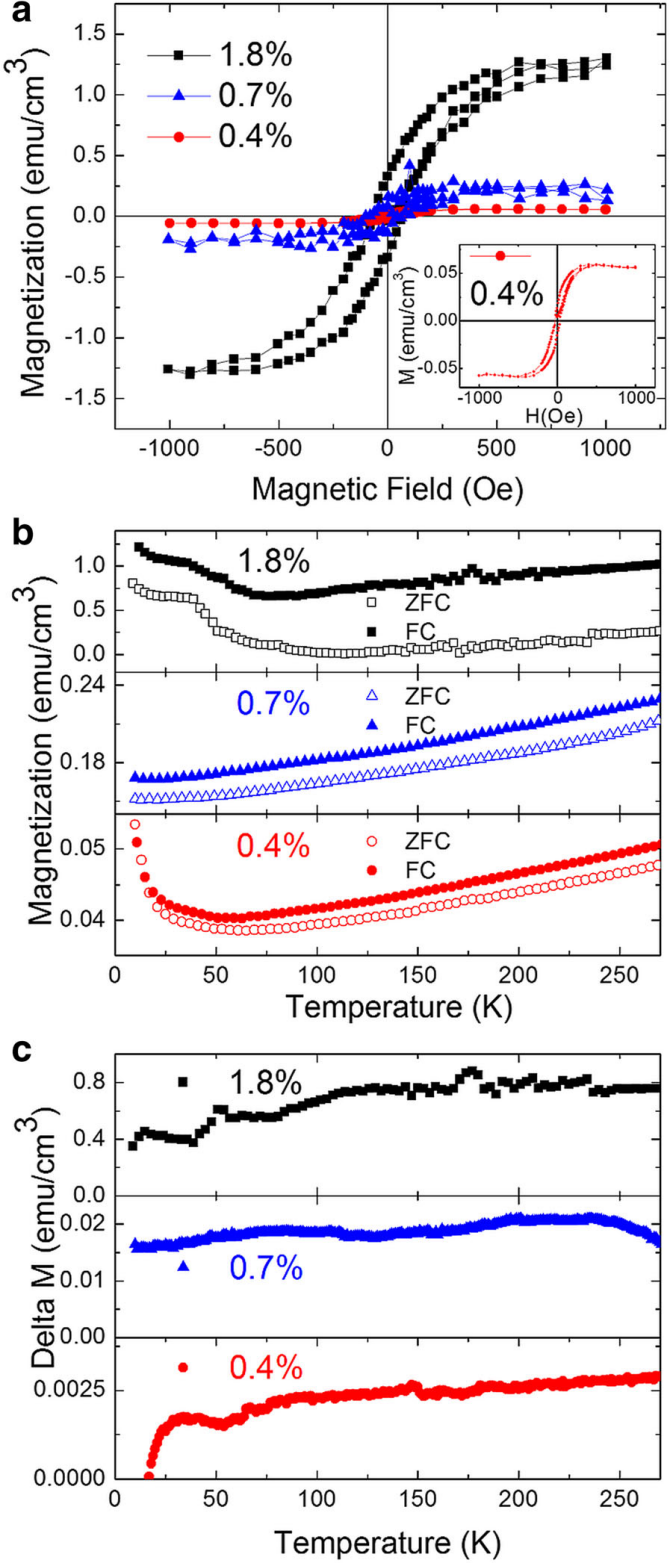
**a** Magnetization versus magnetic field shows hysteresis loop of modulation-doped GaMnN nanorods with average 1.8, 0.7, and 0.4% Mn contents at 350 K by SQUID. The *inset* is the enlarged loop of GaMnN nanorods with an average 0.4% Mn content. Saturation magnetization is much higher on the 1.8% Mn sample than on the 0.4% Mn sample. **b** Magnetization versus temperature ZFC-FC measurements for M-MnNR, S-MnNR, and 40s-MnNR under 2000 Oe in the top, middle, and bottom panels, respectively. **c** Δ*M* versus temperature for the M-MnNR, S-MnNR, and 40s-MnNR samples in the top, middle, and bottom panels, respectively

Investigations of the magnetic properties of modulation-doped GaMnN nanorods were carried out by magnetization versus magnetic field (M vs. H) measurements at room temperature. However, the Mn_2_N_0.86_ has been demonstrated as a weak ferromagnetic material at room temperature besides dominant antiferromagnetism [32, 41]. To avoid the possible ferromagnetism contributed from secondary phases, the modulation-doped GaMnN nanorods without secondary phases were chosen for subsequent magnetic properties studies. In Fig. 9a, the magnetization versus magnetic field curves at 350 K for samples 40s-MnNR, S-MnNR and M-MnNR are plotted. A linear background from the raw data was subtracted to extract the signal from the nanorods. The inset M vs. H shows the enlarged hysteresis effect of GaMnN nanorods with an average 0.4% Mn content. An above-room-temperature ferromagnetism is evidenced by the clear hysteresis loops in all three samples. By adjusting the Mn-doping concentration and the GaN spacers, the saturated magnetization of nanorods increases by almost 25 times indicating a positive correlation with the total spin moment of the material. We also did zero-field-cooled and field-cooled (ZFC-FC) measurements for the single-phase samples by sweeping the temperature and measuring magnetization. These measurements were done at a magnetic field of 2000 Oe. The top, middle, and bottom panels of Fig. 9b show the data for samples M-MnNR, S-MnNR, and 40s-MnNR, respectively. The splitting between the magnetization starts near room temperature and is the strongest for M-MnNR. As the sample is cooled down, magnetization starts to decrease slightly, and at very low temperatures, there is an upturn. This could be due to some paramagnetic signal. An important point to note is the absence of any blocking temperature behavior in the ZFC-FC measurements, implying that samples are free from clusters or secondary phases, corroborating with the XRD and TEM data. The difference in the magnetization (Δ*M* = *M*_FC_ − *M*_ZFC_) is also plotted in Fig. 9c. One can clearly see strong magnetization near room temperature suggesting that the ferromagnetic transition is at room temperature with the strongest signal in M-MnNR.

## Conclusions

The MBE growth and characteristics of modulation-doped GaMnN nanorods have been systematically investigated. The thickness of GaN in each pair of GaN/MnN was varied by the growth time of GaN. A thick GaN spacer with the 40-s growth time between Mn modulation-doped layers reduces the possibility of excess Mn atom aggregation and suppresses the formation of secondary phases effectively. Modulation-doped GaMnN nanorods with varied Mn fluxes were also investigated to achieve single-crystalline GaMnN nanorods with higher Mn content. Samples grown under Mn fluxes of 1.7 × 10^−8^ and 1.9 × 10^−8^ Torr have a Mn concentration of 0.7 and 1.8%, respectively, measured by EDS, and no secondary phases were observed by XRD. Raman scattering indicates the substitution of Ga by Mn atoms. Zero-field-cooled and field-cooled measurements reveal no clusters or secondary phases. Hysteresis loops in magnetization versus magnetic field measurements of GaMnN nanorods show above-room-temperature ferromagnetism, and the saturation magnetizations are enhanced with increasing Mn content which shows a positive correlation. This opens a possible route to applications in nanostructural spintronics.

## Abbreviations

CVD: Chemical vapor deposition
DMSs: Diluted magnetic semiconductors
EDS: Energy dispersive X-ray spectrometry
FESEM: Field emission scanning electron microscopy
HRTEM: High-resolution transmission electron microscopy
HRXRD: High-resolution X-ray diffractometry
LDs: Laser diodes
LEDs: Light-emitting diodes
M vs. H: Magnetization versus magnetic field
NSRRC: National Synchrotron Radiation Research Center
PAMBE: Plasma-assisted molecular beam epitaxy
SQUID: Superconducting quantum interference device
TEY: Total electron yield
XANES: X-ray absorption near edge spectra
ZFC-FC: Zero-field-cooled and field-cooled
